# The Burnout Concept as a Theoretical Framework for Investigating the Caregiving Impact of Relatives of Patients withAddictive Disorders

**DOI:** 10.11621/pir.2022.0307

**Published:** 2022-09-30

**Authors:** Alexandra M. Shishkova, Victor V. Bocharov

**Affiliations:** a V.M. Bekhterev National Research Medical Center for Psychiatry and Neurology, Saint-Petersburg, Russia; b St. Petersburg State Pediatric Medical University, Russia

**Keywords:** Addictive disorders, codependency, informal caregivers, stress, burden, burnout, engagement, methodology

## Abstract

**Background:**

Relatives of patients withaddictive disorders often face significant difficulties in their daily lives. Although the burnout concept is currently considered a significant and promising theoretical framework for studying family members who care for chronically ill patients, its application has encountered considerable difficulties in the area of addiction treatment.

**Objective:**

This article explores the methodology for studying the psychological issues arising in families affected by addictive disorders. We analyzed the social, economic, and cultural conditions of the different study models developed in this field, and identified the difficulties hindering the acceptance of the burnout concept as a theoretical construct for investigation.

**Results:**

There are several main obstacles to the burnout concept’s application to studying the psychology of addictive patients’ families. These obstacles are: 1) a stigmatizing attitude toward the relatives, labelling them as dysfunctional/codependent, or merely passive recipients adjusting to stressful and challenging circumstances; 2) a sole focus on the destructive elements of the “informal caregiver — addicted patient” relationship dynamics; 3) underestimation of relatives’ willingness, experience, and knowledge in the care of their addicted family member and failure to recognize their right to participate in treatment decision-making; and 4) lack of specialized tools for assessing burnout and its opposite pole — the engagement of addicts’ relatives during the patients’ care.

**Conclusion:**

Application of the burnout concept as a theoretical framework allows us to reformulate many psychopathological phenomena described in the family members of addicts, and expands the perspective of psychotherapy by providing the opportunity to conduct interventions to improve relatives’ functioning as caregivers. This, in turn, will contribute to the effectiveness of treatment outcomes for bothaddicts and their families.

## Introduction

Addictive disorders are chronic diseases which often significantly undermine the addict’s physical healthand can have severe psychological and social consequences bothfor the patient himself and for his/her social circle. Most addicts (at least in the initial stages of their disorder) have a family. Family members interact withthe addict to varying degrees, depending on their position or role in the family. Family members usually make attempts to help the patient cope withthe disease and the most involved relatives, who are typically women, experience a greater impact, which results in detrimental effects and suffering for themselves.

Studies have shown that relatives involved in interacting withthe addicted patient typically experience severe anxiety and depression. They may experience helplessness, anger, guilt, or constant concern for the patient’s physical and mental wellbeing. A significant reduction in the quality of their social life, disruption of close family communications, and development of somatic illnesses caused by stress in connection withthe illness of a loved one, are often studied in addicts’ relatives (Copello et al., 2010b; Orford et al., 2005, 2010; Settley, 2020). However, the contribution of a family caregiver to maintaining the patient’s well-being is frequently underestimated due to the attitudes of the professional community which interacts withaddicts’ relatives. These attitudes can be shown by specialists apparently ignoring, or even disapproving of, family care.

In Russia, relatives of patients withaddictive disorders are usually viewed within the framework of the codependency concept. This concept appeared in Russia along withthe Minnesota model of addiction treatment. Currently, various adaptations of the 12-step program, based on the Minnesota model, are being used almost everywhere in clinics engaged in the treatment and rehabilitation of addicted patients. According to the codependency model, the relatives’ care for a patient is perceived as unconscious compensation of their own personal dysfunction. This means that such care has psychological benefits and should be considered as a psychotherapeutic target.

Stress-oriented models, which are common abroad, consider interaction between the family and the addict as a stress factor that can cause strain on family members, leading in some cases to somatic and/or psychic disorders. Relatives’ care in such a context is seen as harmful for their own health, and thus undesirable.

In boththeoretical models currently used for the studying of addicts’ relatives, the principal orientation calls for increasing the distance, or even urging separation, between informal caregivers and addicts. Thus, the very possibility of a caregiving status for the addict’s relatives is questioned.

But actually, “family members are frequently an unpaid and unconsidered resource providing healthand social care to their substance-misusing relatives” (Copello et al., 2010b, p. 67), which defines them as informal caregivers of chronically ill patients.

The term informal caregiver, at the moment, is widely applied in sociology, psychology, and nursing. Its definition varies depending on the application area. Analysis of the literature allows us to give the following definition: an informal caregiver is one who has a social relationship (spouse, parent, child, other relative, neighbor, friend, etc.) withthe care recipient (dependent and in need of care due to various circumstances) and provides unpaid care on an ongoing basis or as required (Shishkova, & Bocharov; 2021a).

The alleged incompatibility of kinship relations withthe caregiver’s role within the framework of the theoretical models used in the field of addictive disorders, on the one hand, and the actual existence of relatives in this role on the other, represent the main contradiction within the current situation, and call for necessary changes.

Analysis of the literature has shown that one of the important principles that currently explains the psychology of informal caregivers is the concept of burnout (Shishkova, & Bocharov; 2021a). Nowadays this concept is not only applied in the occupational context, but is also used in a wider sense to describe “caregiver–care-recipient” relationships, as in the parent–child or “caregiver–chronically ill relative” area (Gérain, & Zech, 2019; Lebert-Charron et al., 2018; de Souza Alves et al., 2019). The burnout concept is described as a considerable and promising theoretical basis for empirical and interventional studies (Gérain, & Zech, 2019). Actively developing research on the burnout observed in caregivers for somatically and mentally ill patients paradoxically has not been applied to the studies of the psychology of addictive patients’ informal caregivers. Thus, the question arises: what prevents the theoretical concept of burnout from being explored in the study of addicts’ caregivers?

As we already mentioned, non-recognition of relatives as caregivers may be one of the reasons for a significant delay in applying the burnout concept in the field of addictive disorders. In present work we analyze the possibilities and barriers of employing the concept of burnout as a theoretical framework for studying the complex psychological problems experienced by relatives taking care of addicted family members. For better understanding of these possibilities and barriers, we first present a brief overview of the burnout concept, its implementation outside the occupational context, and the main tools of its measurement, as well as the theoretical models that are most often used for studying addicts’ relatives.

## Methods

Based on a review of the literature and our own clinical experience, we explored some methodological issues concerning investigation of the caregiving impact of relatives of patients withaddictive disorders. A literature search for publications indexed in Cochrane Library, EMBASE, Web of Science, Scopus, and PsycINFO databases was conducted between 2015 and 2021. The gray literature investigation included searches in databases of theses and manuscripts such as OATD and NDLTD. Additional manual search was based on expert consultations and references in specific journals and books. Articles were analyzed to see if they described the burnout concept and its implementation outside the occupational context, on relatives or families affected by addictive disorders, especially in the aspects of psychological consequences of informal caregiving. The data extraction and following analyses relied on expert knowledge, which allows in-depthunderstanding and synthesis of theories and links between them (Hannes, 2011).

## Results

### The concept of “burnout”

#### Emergence and development of the burnout construct

The phenomenon of ‘burnout’ was first described in the works of Freudenberger (1974) and Maslach (1976). They discovered, independently of one another, that social workers as well as customer service workers often feel emotionally exhausted, and develop negative attitudes toward their clients or patients. These observations served as the basis for the subsequent development of the idea of professional burnout, which has become internationally recognized as a concept to describe this particular type of physical, emotional, and mental exhaustion and stress.

It is important to note that the clinical observations on which the original burnout concept were based came from the experiences of volunteers at the St. Mark’s Free Clinic in New York’s East Village — a free clinic for drug addicts and homeless people. Freudenberger used the term to describe the gradual loss of motivation, lowered commitment, and emotional depletion among the volunteers who were caring for illicit drug abusers (Freudenberger, 1974).

Summing up their observations, Maslach and her colleagues developed a tridimensional concept of burnout: 1) emotional exhaustion; 2) depersonalization; and 3) reduced personal accomplishment. In addition, the authors created the Maslach Burnout Inventory (MBI), which enabled the assessment of an individual’s experience of occupational burnout (Maslach, & Jackson, 1981; 1998).

Emotional exhaustion refers to feelings of being overextended and depleted of one’s emotional and physical resources and is considered a basic component of the syndrome. Depersonalization (cynicism) represents a negative, cynical, or distant attitude toward people encountered in professional life. This negative, excessively detached response to various aspects of the job represents a motivational, interpersonal dimension of burnout. Finally, reduced personal accomplishment reflects a sense of decrease in one’s own competence and effectiveness, and is a self-evaluative dimension of burnout (Maslach, 1998).

The three-factor structure of the concept developed by Maslach was applied unchanged to different professional groups and cultures (Leiter, & Schaufeli, 1996; Schaufeli et al., 2009; Qiao, & Schaufeli, 2011). However, a number of researchers questioned the presence of any common etiology of the emotional exhaustion, depersonalization, and reduced personal accomplishment and claimed that each of the components could develop independently of the others (Golembiewski, & Boss, 1992; Shirom et al., 2005).

The component of reduced personal accomplishment is particularly subject to criticism. A number of empirical studies have demonstrated that the personal accomplishment scale poorly correlates withthe scales of emotional exhaustion and cynicism. Relying on this data, Green, Walkey, and Taylor concluded that exhaustion and cynicism are the core dimensions of burnout (Green et al., 1991; Schutte et al., 2000).

Some other theorists consider burnout a one-dimensional concept (Kristensen et al., 2005; Shirom et al., 2005). According to these authors, exhaustion is the main symptom of burnout; in fact, it is its equivalent.

In recent decades there has been a shift in research focus from burnout towards engagement in work. Although Maslach and Leiter (1999) assumed that engagement and burnout constitute the continuum of work-related well-being, the concept of engagement and its measurement was not sufficiently developed by them. According to Maslach, low scores on the MBI exhaustion and cynicism scales, and a high score on the professional accomplishment scale, reflect engagement phenomena. Subsequent studies have argued that burnout and engagement need to be estimated independently (González-Romá et al., 2006).

At the present time, work engagement is a multidimensional concept. Engagement in work is defined as a positive, fulfilling, work-associated state of mind. It is typically characterized by three features: vigor, dedication, and absorption (Schaufeli et al., 2002). Vigor is characterized by high work capacity and flexible thinking *(i.e.,* creativity) in the work process. Dedication is associated withthe feeling of meaningfulness, enthusiasm, inspiration, and pride. Absorption implies complete concentration, such as when a person is so deeply engrossed withhis/her work that time passes by unnoticed.

For measurement of work engagement, a self-report questionnaire called the Utrecht Work Engagement Scale (Schaufeli, & Bakker, 2003) is widely used. Research has shown that exhaustion and vigor, as well as cynicism and dedication, each constitute their own continuums, which are dubbed energy and identification, respectively (González-Romá et al., 2006).

Analysis of the concept’s current development shows that, in spite of the fact that burnout is well established in psychosocial research and describes a number of important psychosocial problems, its definition and operationalization are still in the process of clarification.

#### Application of the burnout concept in the field of interpersonal relations outside the occupational context

The issue of the applicability of the burnout concept outside the field of professional activity has been discussed for some time. Some authors believe it can only be applied in a work-related context and is uniquely related to occupational exhaustion (Maslach, 1998; Schaufeli et al., 2009). Others describe the phenomenon of burnout as a condition of physical, emotional, and cognitive exhaustion resulting from continued exposure to emotionally straining situations which can occur in various circumstances, not exclusively in a work environment (Pines et al., 1996; Kristensen et al., 2005).

The first papers focusing on the study of burnout as a theoretical concept within the analysis of family relations appeared in studies by Ekberg, Griffith, and Foxall (1986). Their studies appeared to indicate that spouses of chronically ill patients had symptoms similar to those of the burnout that was typical of formal caregivers such as nurses. Three years later, Pelsma (1989) stated that parental burnout also existed and offered an adjusted version of an MBI questionnaire to measure it.

However, more extensive and focused research of informal caregivers’ burnout started after the millennium year 2000 (Norberg, & Green, 2007).

Prolonged disregard for the need to investigate the psychology of the large group of people exposed to severe stress resulting from their taking care of a chronically ill family member and interacting withthem, led to some clear methodological gaps in this field of study. This type of disregard is perhaps to be expected within the usual social and cultural norms of society (Franza et al., 2016). This situation, however, is undergoing significant changes. In particular, the notion that care for a disabled person close to you is a normal duty for a family member that should be performed out of love for them, has been challenged. Also, the belief that care for the disabled relative should be undertaken by a family member, no matter the caregiver’s external circumstances, mental state, or ability to cope, is no longer considered dogma.

There have been sociocultural transformations over recent decades which have significantly affected the institution of the family and the role of women in family interactions. Besides this, the ongoing medico-social process of deinstitutionalization has contributed to observable changes. The transfer of disabled people from institutions, such as hospitals, back to their families and communities to help withthe reduction of inpatient beds and lengths of hospital stays, has heightened the visibility of informal caregivers’ input. In combination, these two tendencies have significantly changed the situation relative to the pressures and social norms of caregiving being the “sacred” duty of a patient’s family, and have produced the need to understand and take into account the efforts and costs of relatives caring for a chronically ill family member.

Currently, the theoretical concept of burnout is widely used in the context of the relationship between caregiver and care-recipient. More precisely, it is used in the study of “parent-child” relations and the relationship between the informal caregiver and the chronically ill patient.

In the field of “parent-child” relations, researchers have been focusing on the problem of parental burnout in the process of a child’s upbringing, and highlighting an increased risk of burnout among parents caring for children suffering from chronic somatic or mental disorders (Roskam et al., 2018; Norberg, & Green, 2007; Lebert-Charron et al., 2018).

At the present time significant data has also been collected on the burnout of informal caregivers who take care of adults withvarious somatic or mental disorders. The results of numerous investigations have shown that the burnout syndrome can negatively affect caregivers’ quality of life and general health, as well as predict the emergence of emotional disorders (Gerain, & Zech, 2019; De Souza Alves et al., 2019).

#### Lack of specialized tools for measuring burnout in the field of interpersonal relations outside the occupational context

These days, when researchers are assessing burnout among family members caring for chronically ill relatives, the vast majority employ adapted versions of the MBI or the Shirom-Melamed Burnout Questionnaire (Lebert-Charron et al., 2018; Norberg, & Green, 2007; de Souza Alves et al., 2019). Bothquestionnaires were originally aimed at studying professional burnout. In the available literature, we found only a few studies that were not solely focused on the adaptation, but on the development of specialized tools for measuring burnout in family relationships (Kmit et al., 2018; Roskam et al., 2018; Shishkova et al., 2021a, b).

Analysis of the measuring tools currently used to diagnose burnout in the field of family relations has revealed a number of significant drawbacks. First is the fact that most of them originally came from the professional arena and were re-purposed for the field of interactions between chronically ill patients and family caregivers. To consider burnout a general condition of the psyche without taking into account its causes does not enable one to consider the specific features of family relations. Direct transfer of the burnout concept and its assessment tools from the professional area to family relationships does not seem to be sufficiently substantiated and thus needs to be adjusted in light of different environments and relationship contexts. Secondly, existing methods of analysis are based on the diagnosis-centered approach, where analysts suggest a set of statements representing symptoms of burnout. The assessment score in these cases only represents the summation of destructive behaviors and processes, without relating them to the resources of the caregiver. This is in contrast to the contemporary view on the concept of burnout that points to the significance of investigating its opposite, engagement.

In the caregiving context, vigor, for example, can represent a sense of adequate energy to solve the issues connected to the treatment and maintenance of the wellbeing of a chronically ill relative. The caregiver’s sense of fulfillment and meaningfulness through their care work can accordingly be considered dedication. So, the development of specialized tools could be considered one of the most vital changes required in research of “engagement-burnout” concepts outside the occupational context. The availability of specialized assessment measures, in turn, will allow study of the conditions and factors that contribute to, or prevent, the informal caregivers’ burnout and engagement.

Thus, the analysis of the current development of the burnout concept and measuring tools used for burnout assessment reveal significant obstacles to the concept’s application to the study of relationships in the families of addicts. These obstacles are primarily related to a certain immaturity of the construct as applied to interpersonal relations outside the occupational context. As we have seen, within the framework of family relations, the burnout construct is focused only on the deficiency component. This limitation determines the inefficacy of the existing assessment tools, as they do not correspond to the burnout concept’s current development. The contemporary burnout-theoretical construct requires the inclusion of engagement, which constitutes the opposite of the burnout pole and reflects constructive personal activity. Another problem is the neglect of the specificity of the interpersonal relationships that arise between the caregiver and care recipient.

The immaturity of the construct as used outside the occupational context, and the methodological inadequacy of the applied tools, act as obstacles to integrating the burnout concept into studies of the psychology of the addictive patient’s family.

### Theoretical models mainly used for studying addicts’ relatives. Challenges of burnout concept integration

#### The concept of codependency

The concept of codependency, which gained popularity in the 1970s, emphasizes the personal deficiencies and destructive behavioral patterns of addicted patients’ relatives. These patterns are considered to interfere withthe therapeutic and rehabilitation process and are to be overcome by breaking pathological (codependent) relationships withthe addicted patient. This approach leads to the disturbed personality and decompensation hypotheses, which were put forward by various authors to interpret some of the psychological characteristics of alcoholics’ wives in the 1950s (Futterman, 1953; Кalashian, 1959).

The development of the codependency concept should be considered within the context of the social circumstances at the time. According to Haaken (1993), it originated from feministic ideas of gender equality and a medical concept focused on the presence of an unhealthy process within addicted patients’ families.

According to feminist ideas, the negative psychological experiences of women having close relationships withaddicts were considered to be caused by gender inequality. The aspects of family living associated withcare and dependence were previously perceived as natural and socially approved. However, in the context of new changing social trends, such relations were considered to be inadequate and unhealthy, resulting in personal and micro-social harm, and therefore needing to be eradicated. Due to this, emotionally close, codependent relationships came to be recognized as at the very least inadequate, or even as an unhealthy situation which needed correction.

G. Dear asserts that the codependency concept is demeaning to women (Dear, 1996) in that it describes behaviors traditionally associated withthe female role in the family, as that of an inadequate and disturbed personality.

In our opinion, this phenomenon reflects the change in gender roles that has been taking place in the Western World. As suggested by Calderwood and Rajesparam (2014), at the time of the initial conceptualization of the codependency model, the stereotype of a woman implied the presence of such qualities as obedience and dependency, and her social activity was usually associated withtaking care of others. The authors believe that nowadays women in Western society are more independent, bothfinancially and emotionally.

As some researchers note, the codependency concept relies on a model that focuses primarily on personal autonomy, which can conflict withthe cultural values of those social systems where an individual’s behavior is determined by family traditions and where an interdependent caretaking style of family relationship is encouraged (Chang, 2012; Granello, & Beamish, 1998; Kwon, 2001).

During the 1990s and beyond, authors began to pay attention to the stigmatizing terminology of the codependency concept and lack of its empirical support for it (Lee, 2014; Dear, 1996; Harper, & Capdevila, 1990; Orford et al., 2013). The use of this pathologizing and stigmatizing terminology when working withaddicts’ family members has a negative effect on their psychological and social functioning (Lee, 2014; Orford et al., 2013). Characterizing women as codependent can significantly damage their self-identification, as this presumes their lack of ability to create mature, realistic, autonomy-based independent relationships. Such a characterization is basically a pseudo-objective social assessment of the personal functioning of women. It is perhaps more traumatizing as it is given by an authority figure, an expert in areas of drugs, alcohol, and other addictive substances. This is replicated numerous times in social environments such as self-help groups, which are ideally meant to provide help.

However, as Lee remarks, while displacement of the research focus away from patient’s family’s role in the development of an addictive disorder, protects the relatives from stigmatization, on the other hand it prevents gaining a coherent view of the family dynamics. This puts much of the blame for what is going on inside the family on the patients themselves (Lee, 2014).

In more recent times, the concept of codependency has become a lot less influential abroad and use of the term itself is becoming increasingly rare. The patients’ relatives are more and more often referred to by terms that do not have such an obvious negative connotation, such as “affected family member,” “concerned family member,” “significant other,” and so on. But in Russia, this theoretical construct still occupies a leading position.

#### Stress-oriented models

Even during the period of the apparent dominance of the codependency concept, there existed alternative approaches to understanding the psychology of the addicted patients’ relatives. At the core of these concepts is, first and foremost, the concept of stress. As early as the 1960-70s, studies were performed which focused on the origin of stress and the reversibility of the disorders that were seen in the addicts’ relatives. In particular, the studies focused on the wives of those suffering from alcoholism (Edwards et al., 1973; Kogan, & Jackson, 1965).

However, the data that did not fit into the pathologizing notion about addict’s relatives’ deficiencies — the methodological basis of codependency — were easily assimilated by the stress concept. This resulted in the codependency construct itself becoming less precise and consequently increasing the number of people whose behavior was described in codependency terms. The most radical opinions expressed by authors who agreed withthis concept declared all those who interacted withthe addict to be codependent, including those who were brought up in a family where one of its members had a marked disorder of a different genesis (Schaef, 1986; Wegscheider-Cruse, 1985).

Currently, the stress-oriented approach is increasingly determining the theoretical comprehension of the psychology of addicts’ relatives internationally. Interaction between a family member and an addict is looked at through the theoretical framework of chronic stress models, where stress, strain, adaptation, and coping serve as the categories analyzed.

Among the evidence-based stress-oriented models widely used in work withaddicts’ relatives, one should mention the Stress-strain-coping-support model (SSCS). UK policy documents report this model to be effective in decreasing drug and alcohol addiction’s negative consequences for society and addicts’ families (Velleman, 2010).

Within this model, interaction between a family and addict is considered a stress factor that can cause strain on family members, leading in some cases to somatic and/ or psychic disorders. In this case, the social support received by the addict’s family serves as a buffer, and such mediation is capable of greatly relieving the negative impact of the addict’s disease (Orford et al., 1998; 2013).

One of the central elements of the SSCS model is the typology of the addict’s relatives’ coping behavior suggested by the authors. In this typology, Orford and his colleagues distinguish three principal different family reactions to the addict’s disease. These include standing up to it, “putting up withit,” and becoming independent. (Orford et al., 2013).

This typology was originally derived from a cross-cultural study of coping behavior strategies of addicts’ relatives carried out by the authors in England and Mexico (Orford et al., 1998). Coping behavior was estimated by Orford and his colleagues according to coping reactions recorded in a previous study. The qualitative and quantitative analysis, and in particular the factor analysis of results achieved by Orford’s team, identified different possible reactions of the relatives living witha drug or alcohol addict. The analysis resulted in identifying three main factors called engagement, tolerant-inactive, and withdrawal (Orford et al., 2010).

It is curious to note that the presented construct is closely associated withinstinctive theories of response to a threat such as “beat,” “standstill,” or “run.” Such a description of how addicts’ relatives cope withthe disease seems to be too simplistic.

The factor the authors called engagement combined strategies representing the family members’ efforts to change the addict’s behavior. This factor included emotional coping — the emotional expression toward the addict; control — the attempt to limit the alcohol and drug use; and assertive reaction — a clear and calm position toward the addiction and support for the addict. A combination of assertive and supporting coping was described by the authors as one reflecting family decisions as to which behavior of addicted family member was acceptable and which was not. Such forms of coping could motivate the addict to stop the substance abuse and/or to seek medical treatment.

According to Orford, tolerance-inactivity factors included strategies such as self-sacrifice and acceptance. Examples include attempts to forgive addicts, inactivity manifested by empty threats, the absence of any demands on the addict, and efforts to keep the peace by any means.

The withdrawal factor which Orford described as bipolar contains strategies aimed at avoidance and independence and represents attempts to keep a distance from the addict by focusing on other interests and needs. Strategies of support are included in this factor witha negative loading (Orford et al., 2010, Velleman, 2010).

While estimating the effectiveness of the interventions based on the SSCS model (known as the 5-Step Intervention), the authors interpreted the total reduction of the coping used by addicts’ relatives as representing positive change. This reduction was primarily due to a decrease in the use of strategies involving active interaction between the family and the addict, such as engagement and tolerance-inactivity (Copello et al., 2000; 2010a; Velleman et al., 2011). In some sampling groups, interventions performed were followed by an increase in withdrawal strategies.

From this we can conclude that spontaneous activity by relatives aimed at fighting the disease and supporting the addict was considered “unhealthy” by the authors (Copello et al., 2000, p. 482). It is important to note that attempts to cope withthe disease may look like self-sacrifice when performed in close family relationships, and that family support can play a significant role in the addict’s recovery.

At the same time, a family member’s increased independence and greater insistence on his or her own needs and rights, which are components of the withdrawal means of coping, are considered by the authors as chiefly positive changes in the dynamic of the caretaking relative–addict relationship.

Such an evaluation is also underpinned by the illustrative material showing the quality analysis data. This is where family members demonstrated how they could withdraw from the addicts regardless of their condition, or even completely separate from them (Copello et al., 2000; 2010a; Velleman et al., 2011). Evidence for the usefulness of the 5-Step Intervention can be seen by the fact that, even when family members considered that their substance-abusing relative had either not improved or had deteriorated, they still showed a significant decrease in symptoms and coping behaviors (Velleman et al., 2011).

The authors conclude that the fewer coping reactions, the more positive changes there are, since most attempts to cope withthe situation, particularly by way of engagement and tolerance, are unhelpful for family members’ experience and health(Copello et al., 2010a; Velleman et al., 2011).

Orford and his colleagues emphasized the multidimensionality of coping strategies and warned against perceiving any of the strategies as dysfunctional or functional without referring them to a specific life situation. But, as we can see, at the core of the considered program is the assumption of absolute personal autonomy as a tool to obtain self-realization. This proposition should sound familiar because of its similarity to the codependency concept.

It is necessary to note that after the 5-Step Intervention, the relatives’ symptom levels had only fallen to the level of those experienced by the sample of psychiatric in-patients and day-patients (Velleman et al., 2011).

One question that arises from this is why the number of symptoms reflecting relatives’ distress is still extremely high if non-adaptive coping strategies such as engagement and tolerance-inactivity associated withpathological symptoms, reflect reduced engagement and emotional detachment from the addicted family member.

We believe that the problem lies in the existing methodological gap. When describing family members’ reactions, Orford et al. consider the addicts’ relatives to be passive recipients adjusting to the stressful conditions of life which are fully determined by the patients’ behavior. At the same time, the relatives’ behavior is constructive at its base, and is aimed at helping the patient recover from the disease. This reflects the relatives’ active role in the transformation of their environment and attempt to eliminate the main source of pathological stress. Underestimation of the relatives’ active position leads to a lack of knowledge about the conditions and the factors associated withthe success or failure of their active involvement in the patient’s life. This subject is considered to be beyond the scope of the concept.

The contradiction between understanding the relative as a sufferer, a passive recipient in stressful circumstances, and the relative’s actual position of a caregiver who gives assistance to his/her addicted relative and struggles withthe illness, creates a methodological gap. This gap requires the implementation of a new special category for resolving existing contradictions. In our view, this could be the notion of engagement within the concept of burnout.

Deprecation of informal caregivers’ efforts, as observed in the field of addiction treatment, can cause a blockage of personally meaningful activities and become one of the potential sources of the addicts’ relatives’ burnout. It is precisely this burnout, in our opinion, that results in the remaining psychopathological symptoms that are described in Orfords’ studies.

In other words, a principal orientation toward separation or withdrawal of relatives from an addicted loved one interferes withthose relatives’ basic emotional needs to provide love and care for someone close to them. To fulfill this need is at least as important for the addicts’ relatives’ self-realization as is the need to achieve abstract personal autonomy. Therefore, today’s core idea of the necessary transformations in patient-caregiver relations should be revised to take into account the need for the active engagement of relatives in the addict’s struggle against the disease, and in maintaining the well-being of an addicted family member.

It is necessary to note that a change in the distance between the chronically ill relative and informal caregiver is not specific for addictive disorders. Increase of the symbiotic tendencies in caregiver/care-recipient relationships is a natural reaction that certainly requires the attention of specialists. However, the presence of symbiotic tendencies is not any reason to deny the constructive personal activity of relatives aimed at supporting chronically ill family members, and cannot be overcome by mechanistically adopting opposite to symbiotic relationship forms of behaviors.

Nevertheless, the important achievement of the stress-oriented approach, compared to the codependency approach, is the partial de-stigmatization of the addicts’ relatives, who are no longer considered dysfunctional and decompensated, but as normal people in a difficult life situation. This results in a significant shift of scientific and research focus toward the experiences, difficulties, and requirements of family members caring for addictive patients.

In the framework of the stress-oriented approach, the family experience of care-giving is also frequently conceptualized as a “burden.” The term “burden of the family” was first mentioned by the American sociologist Treudley (1946) and refers to the consequences for those who care for severely disturbed psychiatric patients.

In psychiatry and somatic clinical practice, empirical studies of the informal caregiver’s “burden” began in the 1960s (Hoenig, & Hamilton, 1966) and are actively ongoing to the present day (Yasuma et al., 2021).

Systematic research of the “burden” of addicts’ relatives only began to be undertaken in the first decade of the 21st century (Biegel et al., 2007). Purposefully studying the difficulties resulting from having an addicted family member, the authors described various conditions, including emotional problems, financial difficulties, stigma, violence, and poor mental health(Di Sarno et al., 2021).

Despite the high relevance of studying and systematizing the difficulties experienced by relatives of patients withaddictive disorders, the theoretical construct of “burden” does not provide a holistic view of the processes in the lives of people involved in interactions withaddicted loved ones. Ignoring the underlying group dynamic relationships of family members and group norms associated withthe socio-cultural context and personal characteristics of caregivers, makes it impossible to form a complete picture so that the necessary psychological assistance can be provided. Additionally, the concept of “burden” is poorly defined and can’t be measured accurately (Gérain, & Zech, 2019; Mosquera et al., 2016).

So, the “burden” concept corresponds to the early stages of developing the burnout concept and focuses only on the negative aspects of caregiver/care-recipient interactions. It can be considered a link in the process of working out a comprehensive methodological framework for studying the addicts’ relatives as informal caregivers. The concept of “burnout” can serve as a theoretical basis for such work.

#### Challenges of burnout concept’s integration into the sphere of addictive disorders

As we have seen, the concept of burnout is considered an important principle in explaining the psychology of family members caring for chronically ill patients. Nevertheless, any research on burnout, besides our own studies (Bocharov et al., 2019; Shishkova et al., 2021a,b,c), has been non-existent in the area of addictive disorders, despite the evidence that family members take an active part in the care of addicts.

In our opinion, the main reason for the lack of studies is a methodological conflict that has not yet been articulated by the scientific community. There is a conflict between the theoretical models which have been used for describing the psychology of addicts’ family members for a considerable time, and the constructs developed to assess the impact of informal caregiving, which have become relevant since the cultural transformations of the last few decades.

In analyzing the described shift in understanding the psychology of addicts’ relatives from codependency models to stress-oriented approaches, we should mention that these changes are due to the influence of various psychotherapeutic paradigms. The popular psychodynamic approach, withwhich the explanatory codependency models are associated, has gradually been replaced by the dominance of the cognitive-behavioral approach. One of the important foundations of this approach is the concept of stress.

The low economic efficiency of psychoanalysis and its branches was one of the factors leading to ignoring the relatives of addictive patients, as they were seen as needing psychotherapeutic care in their own right. During this period, addicts’ relatives organized various self-help groups such as the “Co-dependents Anonymous” community, an organization which has spread throughout the world.

As the cognitive-behavioral approach advanced, psychotherapeutic care became increasingly economically measurable and affordable. This ultimately led to the possibility of its use as the main means of working withthe family members of addicted patients. This was particularly the case in dealing withtheir irrational beliefs and maladaptive coping strategies. Currently, the special psychotherapy provided to relatives of addicted patients in many European countries is conceptual (Velleman, 2010).

Our analysis of the existing approaches to the study of relatives affected by addictive disorders allows us to point out changes in perception and attitude toward such relatives. These changes are reflected in the gradual reduction of stigmatization and the consideration of relatives as normal people in a difficult life situation.

Despite the fundamental difference between the initial message of the stress-oriented models, for example the SSCS, and the codependency concept, there is a significant similarity between the two approaches in understanding the causes of the dysfunction of addicts’ relatives. In bothapproaches the root of the problem is located in their loss of autonomy. Accordingly, the restoration of optimal functioning is achieved in the separation of informal caregiving relatives from the care-recipient patients.

In addition, bothconcepts coincide in postulating the fundamental non-constructiveness of the interaction between the addict and caretaking family members, as well as the tendency to exclude relatives of the addicts from the caregiving process. According to the codependency concept, preoccupation withtheir own problems (personal dysfunctionality) determines the impossibility of family members performing the functions of caregivers. From the standpoint of the stress approach, the vulnerability of addicts’ relatives to the influence of stress also basically excludes them from performing this role. One could agree withthis, but given the fact that the real function of caregiving is performed by relatives, and that the anosognosia typical of addicts often leads to a lack of articulation of the very need for care, the family members are actually the only ones who want to and are able to provide care for their chronically ill relative.

The stigmatizing stereotype in perception and attitude toward addicts’ relatives has substantially determined the deficit-oriented approach currently observed in the field of addiction research and treatment. This approach is characterized by focusing solely on the destructive elements of the “informal caregiver-addicted patient” relationship dynamic, and underestimation of relatives’ willingness, experience, and knowledge in care for their addicted family member. The existing situation causes significant delay in the awareness of the addicted patients’ relatives as informal caregivers and prevents integration of the burnout concept, frequently used for evaluation of caregiving impact, into the field of addictive disorders.

The personal activities of relatives in various forms of care directed to the maintenance of the well-being and fight against the disease of the chronically ill family member, are usually supported by specialists in the somatic and even psychiatric clinics. However, in addiction treatment, they encounter significant resistance. This resistance is associated withthe stereotypes in the evaluation of interactions between the addict and their relatives. This type of relationship is traditionally considered as a dysfunctional and mutually destructive one.

In the somatic clinic, where the existing patient’s illness does not actually depend on the psychological functioning of the family, there is no stigmatizing idea about the impact of relatives on the emergence and maintenance of the pathological process. The term “caregiver” has become widespread here. This term reflects the social shifts that determine the perception of chronically ill patients’ relatives as those performing socially necessary functions, and their participation in treatment and rehabilitation is regarded as a key element in achieving successful results.

The peculiarities of the course of psychiatric diseases, in particular, the need to maintain compliance withmedication and monitor the patient’s condition for their timely hospitalization, have led to acknowledging the importance of engaging families of mentally ill patients as active participants in the therapeutic process. Such engagement is carried out despite the often-expressed stigmatization of this group.

The necessity of providing opportunities for family members to discuss treatment options and acquire awareness and information concerning addiction and the support available is already recognized by addiction service professionals (McDonagh, & Reddy, 2015). However, this should only be considered as an initial step to an effective partnership between relatives and professionals.

For the successful integration of the burnout concept into empirical and interventional studies of the addicts’ relatives, total de-stigmatization of this group of informal caregivers is required; this implies a holistic view that includes not only the dysfunctional aspects, but also a notion of the constructive activity (engagement). In keeping withempowerment ideas, we should recognize the close relatives caring for the addict as equal participants of the treatment process, and enable them to become collaborators in their sick relatives’ treatment decision-making.

## Discussion

In recent years, the concept of burnout has assumed considerable prominence in the research of family members caring for chronically ill patients. By applying this concept, one can systematically examine bothexternal factors — such as the burden relative to the severity and duration of the case — as well as the internal ones. Examples would be the social attitude, self-esteem level, and the degree of symptoms of depersonalization. These factors can determine the effect of giving care on the caregiver. Studying the mechanisms of family caregivers’ burnout, as well as development of prevention programs, are important bothfor caregiver and care-recipient. The chronically ill patient might be left without the assistance of family members because caregiving relatives’ burnout causes them to be negligent. This negligence could cause the patient to succumb to their disease and result in fatality.

The current realities of life, results from continuing deinstitutionalization, increasing life expectancy, and the growthof chronic diseases, make it necessary to support the care provided by relatives of the chronically ill and create conditions for its enhancement and optimization. In this regard, the investigation of informal caregivers’ burnout is an actively developing area, which is reflected in the studies of burnout phenomena in relatives of somatically and mentally ill children and adults (Gerain, & Zech, 2019; Roskam et al., 2018; Norberg, & Green, 2007; Lebert-Charron et al., 2018; De Souza Alves et al., 2019).

Research in the addiction fields is markedly lacking in this respect. In the available literature, our own studies appear to be the only research on burnout in relatives of patients withchemical addictions such as alcoholism, opioid addiction, psychostimulant use disorder, and non-substance addiction such as gambling (Bocharov et al., 2019; Shishkova et al., 2021c). Those investigations were preceded by the development of a theoretical model and specialized assessment tool called the “Level of Relatives’ Emotional Burnout” (Shishkova et al., 2021a,b).

For too long, clinical psychology and addictology have focused solely on the harmful consequences of stress caused by close relations withaddicts and have thus confined the understanding of the behavior of addicts’ relatives to a deficit-oriented model.

A significant sign of coming changes in understanding the interactions between an addict and their relatives is the usage of the term “caregiver,” observed in the literature during last decades (Copello et al., 2010b; Maina et al., 2021). In the field of addictive disorders, the need for the direct supervision of the physical or mental healthof the patients may be less obvious than that of other groups withchronic diseases. However, the severity and consequences associated withthe long-term use of drugs or alcohol may require a strong response from the family members. This would be aimed at ensuring the addict’s physical well-being as well as overcoming the current crisis that the patient is experiencing. Some family members could decide that they have no other option but to take on caring roles, even if they have no desire to (Velleman, 2010).

It is important to note that the reasons for this unwillingness are exhaustion or disappointment in the addict (Velleman, 2010). In our opinion, a similar picture can be observed in the family members of the patients suffering from other chronic diseases and directly reflects the processes associated withburnout.

The use of the term “caregiver” and corresponding theoretical context for understanding the psychological issues arising in the families of addicts are necessary steps to realizing and evaluating the constructive aspect of the activities of addicts’ relatives. In this context, family caregivers are not considered to be obstacles but rather people who assist the professionals in the treatment of the patient.

As researchers in the field of parenting children withchronic illness note, parents bring unique elements to the decision-making process, *i.e.,* “the personal element versus the scientific part” (Jerrett, 1994, p. 1054). Relatives value the healthcare professionals’ expertise. However, they believed that their own experience and knowledge of their child’s care should be recognized too (Coffey, 2006). For the successful achievement оf compliance withtreatment, informal caregivers’ views and contributions need to be taken into account.

The need of the addicts’ relatives for active engagement in treatment and rehabilitation is not sufficiently supported in the framework of current treatment programs. The constructive personal activities of caretaking relatives are reflected, in particular, in their attempts to unite in peer-based social support communities. An example of such an association is the community named “Learn to Cope” (LTC), which was formed in Massachusetts in 2004 (Kelly et al., 2017) in the context of the increasing epidemic of opiate overdose. LTC was organized by a group of parents of drug addicts who shared their experience of fighting against the disease of a loved one. For example, they shared experiences relating to information about various treatment and rehabilitation programs and the difficulties encountered in the process of obtaining insurance for treatment. They invited specialists to conduct psycho-educational and training programs. Unlike groups based on the ideological assumptions of codependency models, an active supportive and protective position of the concerned relative towards the patient is encouraged here (Kelly et al., 2017).

On the one hand, application of the burnout concept as an important methodological principle allows us to reformulate many psychopathological phenomena regarding the family members of addicts. On the other hand, it expands the prospects of psychotherapy by giving an opportunity to conduct interventions improving relatives’ functioning as caregivers.

Re-evaluation and change of the usual image of relatives formed in the field of addiction treatment, opens up the possibility for a new understanding of the phenomena previously described as pathological manifestations of the personality and family disturbance (codependency). We refer to the family members’ cynicism towards the patient, sense of bitterness, hostility, and contempt. Additionally, the relatives’ increasing indifference to the outcome of the disease, the sense of uselessness and helplessness and the decrease of vital energy, in our opinion, should be understood as the result of long-term ineffectiveness of the work of an addict’s informal caregiver to overcome their loved one’s disease in an environment of stigmatization.

Similar phenomena are repeatedly described by various researchers among substance abuse treatment counselors and are interpreted within the framework of the burnout concept (Rapid Response Service, 2019). However, the direct transfer of the symptoms established within the burnout construct in work conditions to the field of the psychology of the addictive patients’ family has seemed to be impractical. This is because it does not take into account the various motives that determine the interaction between informal caregiver and addicted care-recipient. In other words, it is necessary to study how the patterns of burnout manifest themselves in the context of the specific group family dynamics.

## Conclusion

The main goal of the present work was to identify the barriers and benefits of applying the burnout concept in the context of the relationships between addicts and their relatives. We believe that such implementation will contribute to the improvement of current models for studying the psychological issues arising in families affected by additive disorders, and will lead to the development of a deeper understanding of the complex problems and needs being experienced by family members taking care of addicts.

The first obstacle to overcome is the stigmatizing image of patients’ relatives as dysfunctional, passive recipients merely adjusting to the stressful and challenging circumstances. Such an image is now more or less dominant in the majority of theoretical models that serve as the basis for the development of programs used in addiction treatment. The urgent need for a paradigm shift to destigmatize addicts’ relatives does not mean neglecting the specifics of the relationships between the caregiver and the care-recipient, due to the diagnosis-associated picture of the disease. Symbiotic regression manifested in the reduction of the distance between the patient and caregiver is typical for the initial stages of any disease. There is no doubt that such regression can progress and take pathological forms which require correction. This pathological symbiotic relationship has been described in boththe clinical treatment of addictive and mental disorders and has been called codependency and emotional overinvolvement, respectively.

Additionally, approaches that consider the psychology of chronically ill patients’ families from the standpoint of the systems theories of family functioning should not be forgotten. Based on the ideas in these systems models, the presence of the disease in one of the family members may be necessary to maintain the family’s pathological homeostasis. In this situation, the chronically ill family member acts as a patient who has taken on a difficult role for themselves. However, the implementation of a systemic approach requires the involvement of all the patient’s family members in the therapeutic process. This also demands devoting a significant amount of time to reconstruct the family situation before conducting subsequent psychotherapy interventions.

At the present time, clinicians are mainly focusing on reducing the symptoms of informal caregivers’ stress and trauma, and have failed to support their relatives in their beliefs and need to give love and care to chronically ill loved ones. Such beliefs and needs, in turn, could serve as protective factors vis-a-vis caregivers’ burden and burnout. To form a holistic view of the processes in the family of a chronically ill person, including a patient withaddiction, it is necessary to take into account not only such potentially psychopathologizing processes as chronic stress, symbiotic regression, family burden, and burnout. These contribute to the development of the psychological crisis, but it is also important to consider the constructive aspects of the informal caregivers’ personal efforts to overcome the illness and maintain the wellbeing of their relative. This approach will allow us to form a notion about the optimal meaningful activity of the individual, which is necessary to sustain a balance between the desire to provide maximum support to the sick family member on the one hand, and the caregivers need for self-actualization and identity preservation, on the other.

Insufficient research on the constructive personal activity of the addicted patient’s family members under conditions of chronic stress is due to the stigmatizing attitudes that lead to a narrow understanding of the psychology of the addicts’ caregivers. It is also due to the lack of an appropriate methodological framework and ability to measure how the construct of “engagement-burnout” should be quantified.

In conclusion, we would like to say that the authors of this work do not deny the significance of the phenomena described in other conceptual models, but are striving to provide a fuller context for investigation of the current situation in the families of addicts. The lack of a holistic, consistent understanding of what is occurring withinformal caregivers of patients withaddictive disorders often leads to significant distortions in treatment goals and methods, significantly reducing the quality of services provided to the patients and their relatives.
